# Ab initio predictions of structure and physical properties of the Zr_2_GaC and Hf_2_GaC MAX phases under pressure

**DOI:** 10.1038/s41598-021-82402-1

**Published:** 2021-02-05

**Authors:** Muhammad Waqas Qureshi, Xinxin Ma, Guangze Tang, Ramesh Paudel

**Affiliations:** 1 State Key Laboratory of Advanced Welding and Joining, Harbin Institute of Technology, Harbin, 150001 China; 2 School of Materials Science and Engineering, Harbin Institute of Technology, Harbin, 150001 China; 3 Nepal Academy of Science and Technology (NAST), Khumaltar, Lalitpur, 44700 Nepal

**Keywords:** Theory and computation, Mechanical engineering

## Abstract

The electronic structure, structural stability, mechanical, phonon, and optical properties of Zr_2_GaC and Hf_2_GaC MAX phases have been investigated under high pressure using first-principles calculations. Formation enthalpy of competing phases, elastic constants, and phonon calculations revealed that both compounds are thermodynamically, mechanically, and dynamically stable under pressure. The compressibility of Zr_2_GaC is higher than that of Hf_2_GaC along the c-axis, and pressure enhanced the resistance to deformation. The electronic structure calculations reveal that M_2_GaC is metallic in nature, and the metallicity of Zr_2_GaC increased more than that of Hf_2_GaC at higher pressure. The mechanical properties, including elastic constants, elastic moduli, Vickers hardness, Poisson’s ratio anisotropy index, and Debye temperature, are reported with fundamental insights. The elastic constants C_11_ and C_33_ increase rapidly compared with other elastic constants with an increase in pressure, and the elastic anisotropy of Hf_2_GaC is higher than that of the Zr_2_GaC. The optical properties revealed that Zr_2_GaC and Hf_2_GaC MAX phases are suitable for optoelectronic devices in the visible and UV regions and can also be used as a coating material for reducing solar heating at higher pressure up to 50 GPa.

## Introduction

MAX phase materials (with general formula M_n+1_AX_n_ n = 1–3) are the transition metal ternary carbides and nitrides, which provides the bridge between metals and ceramics in terms of properties, and have been attracted significant attention of the scientific community since they were discovered^1^. ‘M’ is denoted as early transition metals, ‘A’ represents the IIIA or IVA elements in the periodic table, and ‘X’ is either carbon or nitrogen. The MAX phases crystallize in P6_3_/mmc hexagonal structure, having the combination of strong covalent M–X bonds and relatively weak metallic M–A bonds, which are responsible for their hybrid properties^2,3^. The unit cell of the MAX phase consists of edge-shared M_6_X octahedra is sandwiched by an A-atom sheet. To be specific, these compounds are machinable, thermal and electrical conductor, damage and irradiation tolerant, corrosion and oxidation resistant, possess high strength and stiffness at high temperature, and having low densities^4–8^. These outstanding attributes make MAX phase materials potential candidates applicable for wear, oxidation, and corrosion resistant coating materials^9–13^, superconducting materials^14^, and the cladding material in a nuclear reactor^15^. Moreover, the MXenes are the 2D derivatives of MAX phases possessing useful application in Li-ion and sodium-ion batteries and supercapacitors^16–18^.

In order to take the full advantages of MAX phase materials in technological applications, a series of experimental and theoretical calculations have been done so far. For example, T. Lapauw et al. synthesized Zr_2_AlC and Hf_n+1_AlC_n_ (n = 1, 2) experimentally, and their lattice parameter was in good agreement with first-principle investigations^19,20^ and Hu et al.^21^ fabricated Nb_4_AlC_3_ by using the spark plasma sintering (SPS) technique and investigated the thermal expansion and electrical conductivity. In addition, Petruhins et al.^22^ predicted the phase stability, and the magnetic state of Cr_2_GaC and thin film of Cr_2_GaC was also prepared using the magnetron sputtering technique, and Hoffman et al.^23^ investigated the neutron irradiation tolerance behavior of Ti_n+1_AC_n_ (A = Al, Si, n = 1, 2) MAX phases and found that their radiation-hard is similar to that of SiC which is mostly used material in nuclear reactors. Moreover, it is experimentally proved that Al-based MAX phase materials are excellent oxidation resistant as bulk material and thin-film form because a protective Al_2_O_3_ layer formed, which further hinders the oxidation of core material^9,24^. The theoretical research on the MAX phase material is mainly based on density functional theory (DFT)^25^ calculations, and numerous studies can be found in literature^26,27^.

The theoretical studies related to MAX phase materials are enormous, and it's growing abruptly compared to experimental work. Recently, the electronic, elastic, thermodynamic, and vibrational properties of M_2_GaC MAX phases with M = Ti, Mo, V, Nb, Mn, and Cr have been studied theoretically^14,22,28–31^. Romeo et al. investigated the structural, elastic, and electronic properties of Nb_2_AC (where A = Sn, In, and S) under pressure range from 0 to 10 GPa and found the linear compressibility of unit cell along the c axis compared with the increase in pressure^32,33^. Similarly, Bouhemadou et al. investigated the structural, electronic, and elastic of a wide range of M_2_AX phases^34–36^, and pressure effect on structural and elastic properties was comparatively studied^37–39^. Among numerous computed investigations, the M_2_GaC (M = Zr, Hf) MAX phases are the least studied 211-type MAX phases^26,40,41^. Recently, the structural stability, elastic, phonon, and thermodynamic properties of Zr_2_GaC and Hf_2_GaC have been studied^42^ and computed elastic properties are in excellent agreement with the available data^43^. However, the properties of Zr_2_GaC and Hf_2_GaC MAX phases need to be studied under high pressure, and the stability of these compounds with respect to their competing phases should be explored further.

These above mentioned experimental and theoretical studies motivated us to investigate the electronic structure, mechanical, dynamical, and optical characteristics of Zr_2_GaC and Hf_2_GaC MAX phases under high pressure. In the present work, the structural stability, electronic, mechanical, phonon, and optical properties of the M_2_GaC (M = Zr, Hf) MAX phase materials have been investigated under pressure ranging from 0 to 50 GPa using the first-principles plane-wave pseudopotential DFT within the generalized gradient approximation (GGA). The results showed that the M_2_GaC MAX phases are electronically, elastically, and optically anisotropic in nature and suitable for high-temperature application, coating material, and optoelectronic devices. The paper is organized as follows: detailed computational methods is second section, the obtained results and discussion of M_2_GaC are presented in third section, and in fourth section summary of this research is given.

## Computational details

The Density Functional Theory (DFT) is an ideal quantum mechanical tool to determine the ground state properties and electronic structures of the molecules and solid materials^44^. The calculations presented in this work were performed using the Cambridge Serial Total Energy Package (CASTEP) based on DFT. The exchange–correlation was treated within the generalized gradient approximation (GGA) of Perdew–Burke–Ernzerhof (PBE)^45^ with the plane-wave ultrasoft pseudopotential code. Exchange correlation in GGA is a function of charge density ρ(r) and spatial gradient and empirically written as; $E_{XC}^{GGA} = \int {\varepsilon_{XC} F\left( {\rho ,\nabla \rho } \right)} d^{3} r$. The accuracy of calculations depends on two parameters, i.e., first, the kinetic energy cut-off, which determines the number of plane waves in the expansion, and second, the special k-points used for the Brillouin zone (BZ) integration. For both M_2_GaC (M = Zr, Hf) MAX phases, plane waves cut off energy was set at 600 eV, and the Monkhorst-pack^46^ scheme of 15 × 15 × 3k-points was employed, respectively. The ground state structural parameters were determined using Broyden–Fletcher–Goldfarb–Shanno (BFGS)^47^ minimization technique while the convergence tolerance were set as follows: total energy tolerance less than 5 × 10^–6^ eV/atom, stress component less than 0.02 GPa, maximum force tolerance 0.01 eV/Ǻ, and maximum displacement of the atom during the geometric optimization was less than 0.0005 Ǻ. Finally, Debye’s temperature, mean, transverse, and longitudinal sound velocities were calculated using elastic constants. For the dynamical stability of M_2_GaC MAX phases, the phonon dispersion was computed using a finite displacement method implemented in Material Studio^48^. The crystal orbital Hamilton population (COHP) calculations are performed using an open-source Local Orbital Basis Suite Towards Electronic-Structure Reconstruction (LOBSTER) code to investigate the chemical bonding^49–51^.

The enthalpy of formation of M_2_GaC phase was calculated through the linear optimization procedure as:

1 $$\Delta H_{cp} = E_{211} - E_{{competing{ - }phases}}$$

where $H_{211}$ is the enthalpy of the 211-M_2_GaC MAX phases and $H_{{competing{ - }phases}}$ is the total enthalpy of the set of competing phases.

The energy of formation per atom $\left( {\mathop E\nolimits_{for}^{{M_{2} GaC}} } \right)$ for M_2_GaC MAX phases can be calculated as^52^:

2 $$\mathop E\nolimits_{for}^{{M_{2} GaC}} = \frac{{\mathop E\nolimits_{total}^{{M_{2} GaC}} - \left( {x\mathop E\nolimits_{solid}^{M} + y\mathop E\nolimits_{solid}^{Ga} + z\mathop E\nolimits_{solid}^{C} } \right)}}{x + y + z}$$

where $E_{total}^{{M_{2} GaC}}$, $E_{solid}^{M}$, $E_{solid}^{Ga}$, and $E_{solid}^{C}$ are the total energy of M_2_GaC MAX phase, M, Ga, and C atoms in the solid form, and x, y, z is the number of atoms for M, Ga, and C elements in the unit cell, respectively.

The distortion parameter for octahedra ($o_{r}$) and that of trigonal prism ($p_{r}$) can be defined as follows^53,54^:

3 $$\mathop o\nolimits_{r} = \frac{\sqrt 3 }{{2\sqrt {4z^{2} \left( \frac{c}{a} \right)^{2} + \frac{1}{12}} }}$$

4 $$\mathop p\nolimits_{r} = \frac{1}{{\sqrt {(0.25 - z)^{2} \left( \frac{c}{a} \right)^{2} + \frac{1}{3}} }}$$

The DOS at Fermi level is used to investigate the metallicity at ambient temperature using following expression^55,56^:

5 $$f_{m} = \frac{{n_{m} }}{{n_{e} }} = \frac{{k_{B} T \times N\left( {E_{F} } \right)}}{{n_{e} }} = \frac{{0.026 \times N\left( {E_{F} } \right)}}{{n_{e} }}$$

where $n_{m}$ is the thermally excited number of electrons and $n_{e}$ is the total number of valence electrons in the unit cell. $k_{B}$ and $N\left( {E_{F} } \right)$ are the Boltzmann constant, and value of DOS at Fermi level in unit states/eV/unit cell, respectively.

The Fermi energy of M_2_GaC MAX phases are used to estimate the velocity of electron ($v_{F}$) near the Fermi level:

6 $$v_{F} = \sqrt {\frac{{2E_{F} }}{m}}$$

Then use this value to estimate the conductivity (σ) as:

7 $$\sigma = \frac{{ne^{2} \tau }}{m} = \frac{{ne^{2} l}}{{mv_{F} }},\tau = \frac{l}{{v_{F} }}$$

where $\tau$, *m, n, e*, and *l* are the time between two collisions, mass of electron, number of electrons, electron’s charge, and mean free path of electron, respectively.

To calculate the bulk, shear, and Young’s modulus, the following equations used within the Voigt (V)^57^, Russ (R)^58,59^, and Voigt-Russ and Hill (VRH)^60,61^ approximation scheme:

8 $$B_{V} = \frac{1}{9}\left( {2\left( {C_{11} + C_{12} } \right) + 4C_{13} + C_{33} } \right)$$

9 $$B_{R} = \frac{{\left( {\left( {C_{11} + C_{12} } \right)C_{33} - 2C_{12}^{2} } \right)}}{{\left( {C_{11} + C_{12} + 2C_{33} - 4C_{13} } \right)}}$$

10 $$G_{V} = \frac{1}{30}\left( {C_{11} + C_{12} + 2C_{33} - 4C_{13} + 12C_{44} + 12C_{66} } \right)$$

11 $$G_{R} = \frac{{\frac{5}{2}\left[ {\left( {\left( {C_{11} + C_{12} } \right)C_{33} - 2C_{12}^{2} } \right)^{2} } \right]C_{55} C_{66} }}{{\left[ {3B_{V} C_{55} C_{66} + \left( {\left( {C_{11} + C_{12} } \right)C_{33} - 2C_{12}^{2} } \right)^{2} \left( {C_{55} + C_{66} } \right)} \right]}}$$

12 $$B = \frac{1}{2}\left( {B_{V} + B_{R} } \right)$$

13 $$G = \frac{1}{2}\left( {G_{V} + G_{R} } \right)$$

where Bv, Gv, and Br, Gr are the bulk and shear modulus in terms of Voigt and Russ approximation, respectively. The Values of Young’s modulus and Poisson’s ratio obtained by:

14 $$E = \frac{9BG}{{3B + G}}$$

15 $$\sigma = \frac{3B - 2G}{{2\left( {3B + G} \right)}}$$

The mechanical Anisotropy (A) calculated as follows:

16 $$A = \frac{{4C_{44} }}{{C_{11} + C_{33} - 2C_{13} }}$$

To calculate the hardness, the semi-empirical method based on Pugh’s ratio, which is proposed by the Chen et al.^62^, was adopted as:

17 $$H_{v} = 2\left( {k^{2} G} \right)^{0.585} - 3$$

where k is the Pugh’s ratio (G/B), and G is the shear modulus. The Debye temperature determined by the Anderson method^63^:

18 $$\theta_{D} = \frac{h}{{k_{B} }}\left[ {\frac{3n}{{4\pi V_{a} }}} \right]^{\frac{1}{3}} V_{m}$$

The transverse sound velocity (*V*_*t*_), longitudinal sound velocity (*V*_*l*_), and mean sound velocity (V_m_) calculated by:

19 $$V_{m} = \left[ {\frac{1}{3}\left( {\frac{2}{{V_{t}^{3} }} + \frac{1}{{V_{1}^{3} }}} \right)} \right]^{{ - {\raise0.7ex\hbox{$1$} \!\mathord{\left/ {\vphantom {1 3}}\right.\kern-\nulldelimiterspace} \!\lower0.7ex\hbox{$3$}}}} ,\;{\text{with}}\;V_{t} = \left( {\frac{G}{\rho }} \right)^{\frac{1}{2}} ,\;{\text{and}}\;V_{1} = \left( {\frac{3B + 4G}{{3\rho }}} \right)^{\frac{1}{2}}$$

where *h* is Planck’s constant, *k*_*B*_ is Boltzmann’s constant, *n* represents the number of atoms per unit cell, *V*_*a*_ is the atomic volume, respectively. The melting temperature (T_m_) of MAX phase materials having hexagonal crystal structure was calculated from the elastic constants as follows^64^:

20 $$T_{m} = 3C_{11} + 1.5C_{33} + 354$$

The 3D Young’s modulus surface was obtained using the following equation^65^:

21 $$\frac{1}{E} = S_{11} \left( {\mathop l\nolimits_{1}^{4} + \mathop l\nolimits_{2}^{4} + 2\mathop l\nolimits_{1}^{2} \mathop l\nolimits_{2}^{2} } \right) + S_{33} \mathop l\nolimits_{3}^{4} + \left( {S_{44} + 2S_{13} } \right)\left( {\mathop l\nolimits_{1}^{2} + \mathop l\nolimits_{2}^{2} } \right)\mathop l\nolimits_{3}^{2}$$

where S_ij_ and l_i_ represent the elastic compliance tensor of M_2_GaC MAX phases and direction cosine in the sphere coordination, respectively.

The value of the imaginary part can be calculated from the moment matrix element between the occupied and unoccupied electronic states as:

22 $$\varepsilon_{2} \left( \omega \right) = \frac{{2e^{2} \pi }}{{\Omega \varepsilon_{0} }}\sum\limits_{k,v,c} {\left| {\psi_{k}^{c} \left| {u.r} \right|} \right.\left. {\psi_{k}^{v} } \right|}^{2} \delta \left( {E_{k}^{c} - E_{k}^{v} - E} \right)$$

where e is the electronic charge, $\omega$ is the light frequency, u is the vector defining the polarization of the incident electric field, $\psi_{k}^{c}$ and $\psi_{k}^{v}$ are the conduction and valence band wave function at k, respectively.

## Results and discussion

### Structural properties and compressibility

**Figure 1:**
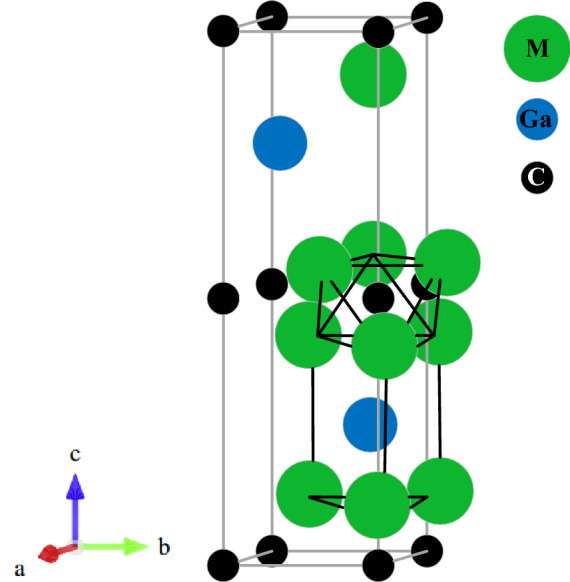
The unit cell of the M_2_GaC MAX phase (M = Zr, Hf). An edge shared [M_6_X] octahedra and a [M_6_A] trigonal prism are outlined.

The considered M_2_GaC (M = Zr, Hf) MAX phase crystallizes in the hexagonal structure with a space group P6_3_/mmc (No. 194) in which M_6_C (M = Zr, Hf) edge shared octahedron is interleaved by atomic layer of Ga atom. There are eight atoms per unit cell in MAX phase compounds, and the unit cell contains two formula units. The atoms in the M_2_GaC MAX phase are placed as follows: C atoms are at 2a (0, 0, 0), Ga atoms are at 2d (2/3, 1/3, 1/4), and M atoms are at 4f. (1/3, 2/3, z_M_), respectively where z_M_ is known as the internal parameter. Figure 1 shows the optimized unit cell of the M_2_GaC MAX phase at 0 GPa pressure. The optimized parameters for each pressure up to 50 GPa with the pressure step of 10 GPa is tabulated in Table 1. With the increase in pressure, the lattice parameters, unit cell volume reduced while the internal parameter increases for both the MAX phases studied.

**Table 1:** Calculated lattice parameters (a) and (c) in Å, unit cell volume (A^3^), c/a, internal parameter (z), no. of DOS at E_F_ (states/eV/unit cell), formation energy *E*_*for*_ (eV/atom), metallicity (*f*_*m*_), and Fermi energy E_F_ (eV) for M_2_GaC MAX phase (M = Zr, Hf) obtained by GGA-PBE at 0–50 GPa pressure.

M_2_GaC	Pressure (GPa)	a (Å)	c (Å)	V (Å ^3^)	c/a	*z*	*N *(*E*_*F*_)	*E*_*F*_	*E*_*for*_	*f*_*m*_ (× 10^–3^)	References
Zr_2_GaC	0	3.330	14.257	136.92	4.28	0.0882	3.00	2.19	− 7.59	5.20	^42^
	10	3.261	13.881	127.84	4.256	0.0907	3.11	2.27	− 6.55	5.39	
	20	3.206	13.619	121.26	4.247	0.0922	3.31	2.39	− 5.58	5.73	
	30	3.160	13.422	116.08	4.247	0.0932	3.57	2.47	− 4.66	6.18	
	40	3.119	13.258	111.73	4.250	0.0941	3.99	2.53	− 3.77	6.91	
	50	3.082	13.121	107.93	4.257	0.0949	4.27	2.59	− 2.91	7.40	
Hf_2_GaC	0	3.324	14.025	134.20	4.219	0.0907	2.47	2.22	− 7.45	4.20	^42^
	10	3.262	13.706	126.36	4.201	0.0928	2.49	2.31	− 6.43	4.31	
	20	3.213	13.470	120.47	4.191	0.0943	2.49	2.39	− 5.47	4.31	
	30	3.172	13.282	115.76	4.186	0.0955	2.52	2.45	− 4.55	4.36	
	40	3.135	13.134	111.85	4.188	0.0965	2.57	2.51	− 3.66	4.47	
	50	3.103	13.007	108.50	4.191	0.0973	2.61	2.56	− 2.80	4.52	

The thermodynamic stability of M_2_GaC MAX phase materials is predicted in terms of formation enthalpy $\left( {\Delta H_{cp} } \right)$ by comparing the total energy M_2_GaC MAX phase to the energy of non-MAX competing phases (single elements, binary and ternary compounds). Table 2 shows the most competing phases considered for M_2_GaC MAX phases determined by using linear optimization procedure^66^. This linear optimization procedure has been successfully used for predicting many MAX phases^67,68^ in which a phase is considered to be stable if $\Delta H_{cp} < 0$. Based on identified competing phases, the Eq. (1) for Zr_2_GaC and Hf_2_GaC MAX phases can be rewritten as:

$$\Delta H_{cp} = E\left( {Zr_{2} GaC} \right) - E\left( {Zr_{2} Ga} \right) - E\left( C \right) < 0$$

$$\Delta H_{cp} = E\left( {Zr_{2} GaC} \right) - E\left( {ZrC} \right) - E\left( {Ga} \right) - E\left( {Zr} \right) < 0$$

$$\Delta H_{cp} = E\left( {Hf_{2} GaC} \right) - E\left( {Hf_{2} Ga} \right) - E\left( C \right) < 0$$

**Table 2:** Lattice parameter, unit cell volume, and total energies of M_2_GaC and its competing phases considered. The phases in bold are the non-MAX competing phases obtained from the linear optimization to calculate the formation enthalpy of the M_2_GaC MAX phase.

Phase	Space group	a (Å)	b (Å)	c (Å)	V (Å ^3^)	E (eV/fu)
**C**	**P6**_**3**_**/mmc (194)**	**2.467**	**2.467**	**8.685**	**45.80**	− **155.09682**
C	FD-3M (227)	3.573	3.573	3.573	45.64	− 154.96104
Ga	I4/mmm (139)	2.797	2.797	4.863	38.06	− 1024.7819
Ga	Cmce (64)	4.530	7.751	4.592	161.29	− 1026.434
**Ga**	**R-3m (166)**	**9.142**	**9.142**	**17.45**	**1253.32**	− **684.27497**
Ga	I-43d (220)	6.088	6.088	6.088	225.71	− 1026.4272
Ga	Cmcm (63)	4.676	7.832	4.608	168.76	− 1021.2854
Zr	P63/mmc (194)	3.239	3.239	5.172	46.99	− 1281.0882
Zr	Im-3m (229)	3.583	3.583	3.583	46.008	− 640.49715
**Zr**	**Fm-3m (225)**	**4.536**	**4.536**	**4.536**	**93.37**	− **320.26041**
Zr	Ibam (72)	5.499	6.200	8.32	283.73	− 554.54599
Zr	P6/mmm (191)	2.917	2.917	7.6	56.04	− 1273.7137
Hf	Im-3m (229)	3.541	3.541	3.541	44.42	− 204.33206
Hf	P6_3_/mmc (194)	3.198	3.198	5.075	44.96	− 408.84959
Hf	Fm-3m (225)	4.481	4.481	4.481	90.003	− 102.19336
Hf	P6/mmm (191)	5.003	5.003	3.087	66.94	− 408.7968
GaC	P63mc (186)	3.262	3.262	5.61	51.71	− 1102.9535
ZrC	Pm-3m (221)	2.934	2.934	2.934	25.27	− 717.70793
ZrC	F-43m (216)	5.106	5.106	5.106	133.13	− 179.58292
ZrC	P6_3_/mmc (194)	3.391	3.391	5.29	52.69	− 718.78154
**ZrC**	**Fm-3m (225)**	**4.724**	**4.724**	**4.724**	**105.44**	− **179.73671**
HfC	Fm-3m (225)	4.651	4.651	4.651	100.63	− 70.667078
HfC	Pm-3m (221)	2.896	2.896	2.896	24.29	− 281.2927
HfC	F-43m (216)	5.042	5.042	5.042	128.22	− 70.487579
HfC	P-6m2 (187)	3.23	3.23	2.916	26.36	− 281.90457
HfC	P6_3_/mmc (194)	3.342	3.342	5.202	50.34	− 282.47912
ZrGa	I41/AMD (141)	10.637	10.637	10.637	153.565	− 1667.604
**Zr**_**2**_**Ga**	**I4/mcm (140)**	**6.706**	**6.706**	**5.485**	**246.7**	− **769.40564**
Zr_3_Ga_2_	P4/mbm (127)	7.377	7.377	3.706	201.70	− 1590.3401
Zr_3_Ga	Pm-3m (221)	4.367	4.367	4.367	83.31	− 589.74576
ZrGa_2_	Cmmm (65)	4.016	13.01	4.157	217.32	− 898.08672
ZrGa_3_	I4/mmm (139)	3.993	3.993	17.66	281.60	− 930.20393
HfGa	PBCM (57)	9.171	8.503	5.648	440.437	− 1231.2989
**Hf**_**2**_**Ga**	**I4/mcm (140)**	**6.712**	**6.712**	**5.291**	**238.39**	− **478.63285**
Hf_3_Ga	I4/mmm (139)	4.205	4.205	9.18	162.41	− 410.03684
HfGa_3_	I4/mmm (139)	3.902	3.902	9.091	138.48	− 821.12364
**Zr**_**2**_**GaC**	**P6**_**3**_**/mmc (194)**	**3.330**	**3.330**	**14.257**	**136.92**	− **1193.291**
**Hf**_**2**_**GaC**	**P6**_**3**_**/mmc (194)**	**3.324**	**3.324**	**14.025**	**134.20**	− **757.052**

Both the Zr_2_GaC and Hf_2_GaC phases fulfill the criterion $\Delta H_{cp} < 0$, indicating that the M_2_GaC MAX phases are thermodynamically stable and can be formed experimentally. Moreover, the calculated formation energy per atom for Zr_2_GaC and Hf_2_GaC at 0 GPa is − 7.59 eV/atom and − 7.45 eV/atom, respectively. There has been a increase in energy of formation for Zr_2_GaC and Hf_2_GaC as pressure is increased.

**Figure 2:**
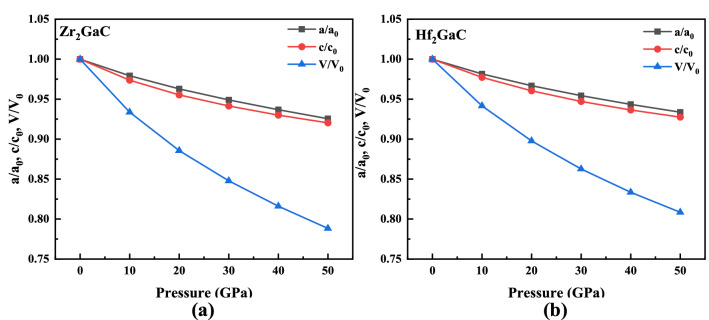
The pressure dependence of ${\text{a}}/{\text{a}}_{0}$, c/${\text{c}}_{0}$ and volume V/V_0_ for M_2_GaC (M = Zr, Hf) MAX phase at T = 273 K.

**Figure 3:**
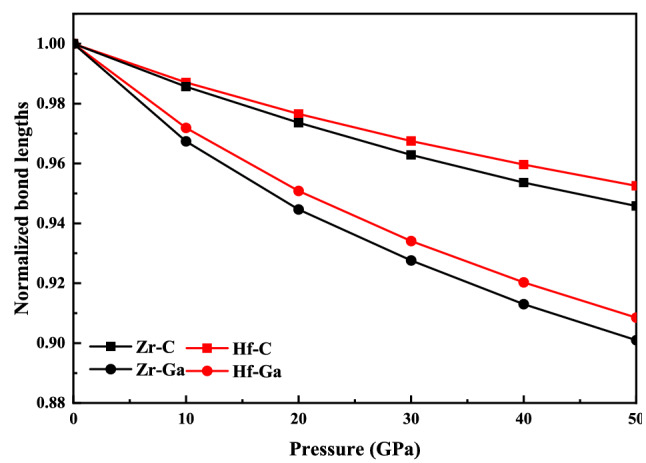
Variation of normalized bond lengths between M–Ga and M–C atoms of M_2_GaC (M = Zr, Hf) MAX phase with pressure.

Figure 2 shows the effect of pressure on the normalized lattice parameters $$a/a_{0}$$, c/$$c_{0}$$ and volume V/V_0_ ($$a_{0}$$, $$c_{0}$$, and V_0_ are the lattice parameters and volume at the 0 GPa pressure). The compressibility for both the M_2_GaC MAX phase along the c-axis is more than that of the a-axis as pressure increases from 0 to 50 GPa^32,33^. Similarly, the volume change ratio gradually (V/V_0_) decreases with an increase in pressure, indicating that the compressibility of the M_2_GaC MAX phase system is strong. The compressibility results are in good agreement with other Zr and Hf based MAX phases^37,39,69^. Moreover, the V/V_0_ of Zr_2_GaC and Hf_2_GaC was reduced to 21.1% and 19.1%, respectively. In other words, external pressure has a more significant effect on Zr_2_GaC than the Hf_2_GaC MAX phase. Zhao et al.^70^ studied the properties of Ti_3_AC_2_ (A = Al, and Si) MAX phases under pressure in which the ratio of V/V_0_ of Ti_3_AlC_2_ and Ti_3_SiC_2_ was reduced to 18.7% and 16.9%, respectively. It is worth noticing that at high pressure, the volume ratio curve becomes steady, indicating that change in atomic distance is smaller, which results in stronger mutual repulsion as atoms come further closer; eventually, compression of the crystal becomes more difficult. Similarly, the reduction in lattice parameter ratios ($$a/a_{0}$$, c/$$c_{0}$$) are in the order of Zr_2_GaC > Hf_2_GaC. Figure 3 exhibits the normalized bond lengths $${\raise0.7ex\hbox{${l_{1} }$} \!\mathord{\left/ {\vphantom {{l_{1} } {l_{10} }}}\right.\kern-\nulldelimiterspace} \!\lower0.7ex\hbox{${l_{10} }$}}$$ and $${\raise0.7ex\hbox{${l_{2} }$} \!\mathord{\left/ {\vphantom {{l_{2} } {l_{20} }}}\right.\kern-\nulldelimiterspace} \!\lower0.7ex\hbox{${l_{20} }$}}$$ (where $$l_{10}$$ and $$l_{20}$$ are the bond lengths of M–Ga and M–C at 0 GPa, respectively) M–Ga and M–C atoms within the M_2_GaC (M = Zr, Hf) MAX phase unit cell versus pressure. It can be noted that the bond length M–Ga (M = Zr, Hf) becomes steeper than that of M–C, indicating that the direction along the M–Ga is easily compressed compared to the M–C bond. These results agreed with a weaker metallic bond between M–Ga atoms in the unit cell, which defines the lattice parameter c. The bond lengths of Zr–Ga and Zr–C reduced more than Hf–Ga and Hf–C, exhibiting that the Zr_2_GaC MAX phase is more compressible than Hf_2_GaC along the Zr–Ga direction.

Furthermore, the c/a and the internal parameter z were used to calculate the distortion within the structure. As mentioned earlier that the crystal structure of the MAX phase is hexagonal and constituted by [M_6_X] octahedron and [M_6_A] trigonal prism. For an ideal structure, the octahedra and trigonal parameters should be equal to one ($o_{r}$ = $p_{r}$ = 1), the variation of $o_{r}$ and $p_{r}$ from 1 tells the distortion in these polyhedra. Moreover, Aydin et al. proposed that the smaller the distortion, the more stable the structure is^71^. In this work, the distortion parameters deduced from the optimized lattice parameters for M_2_GaC MAX are: $o_{r}$ = 1.071 and $p_{r}$ = 1.109 for Zr_2_GaC, and $o_{r}$ = 1.058 and $p_{r}$ = 1.128 for Hf_2_GaC, respectively. This indicates the distortion in the octahedra and trigonal prism in both proposed MAX phase structures and the distortion is small and similar because $o_{r}$/$p_{r}$ = 0.93–0.97. This behaviour is explained as steric effect^72^. The reported $o_{r}$ and $p_{r}$ for Ti_2_GaC MAX phase are 1.088 and 1.081 with $o_{r}$/$p_{r}$ = 1.00^53^. It can be seen that the octahedra and trigonal prisms’ distortion in Ti_2_GaC is smaller than that of Zr_2_GaC and Hf_2_GaC MAX phases. Thus, it is concluded that the structure of Ti_2_GaC is more stable than the M_2_GaC (M = Zr, Hf) MAX phases. The distortion parameters for IV-B and V-B group transition metal MAX phases are deduced from their optimized lattice parameters in the literature for comparison^38,53^. We found that, with increasing the valance of M element (Ti $\to Zr \to Hf$, and V $\to Nb \to Ta$), $o_{r}$ decreases towards value 1, but $p_{r}$ increases to values higher than 1 in M_2_GaC (See Table 3). In other words, increasing the valance of M element in M_2_GaC MAX phases, distortion in octahedra decreases and tends to be stable while distortion in trigonal prisms increases. The distortion in [M_6_X] octahedron and [M_6_A] trigonal prisms can be observed in other MAX phases with different A-site elements^26^.

**Table 3:** The distortion parameters of octahedra (${\text{o}}_{{\text{r}}}$) and trigonal prisms (${\text{p}}_{{\text{r}}}$) of M_2_GaC (M = IV-B and V-B group transition metals) MAX crystal structure.

M_2_GaC	$o_{r}$	$p_{r}$	References	M_2_GaC	$o_{r}$	$p_{r}$	References
Ti_2_GaC	1.088	1.081	^53^	V_2_GaC	1.075	1.086	^38^
Zr_2_GaC	1.071	1.109	This work	Nb_2_GaC	1.046	1.109	^38^
Hf_2_GaC	1.058	1.128	This work	Ta_2_GaC	1.042	1.121	^38^

### Electronic properties

**Figure 4:**
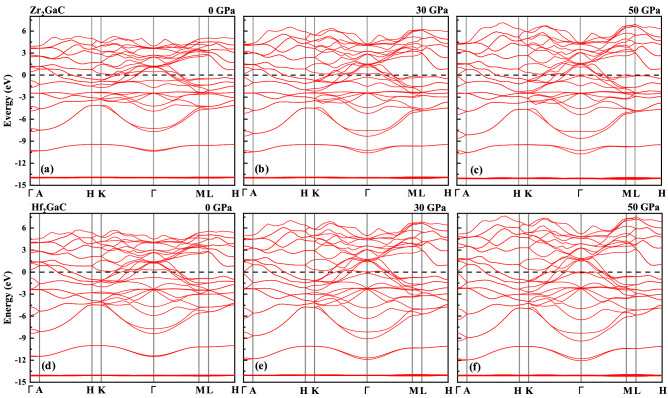
Band structure for Zr_2_GaC at **a** 0 pressure, **b** 30 GPa, and **c** 50 GPa, and for Hf_2_GaC at **d** 0 pressure, **e** 30 GPa, and **f** 50 GPa. Fermi level is set to 0 eV.

The band structure for the M_2_GaC MAX phase at equilibrium lattice parameter within the GGA-PBE was calculated and discussed. Figure 4 shows the band structures from − 15 to 8 eV energy range along the high symmetry lines of the Brillouin zone (Γ–A–H–K–Γ–M–L–H) at 0, 30, and 50 GPa. The conduction bands overlap with the valance band at the Fermi level without having a bandgap in the vicinity of the Fermi level resulting in the metallic behavior of the M_2_GaC MAX phase compounds. Moreover, band structures' appearance resembles other metallic MAX phases, i.e., Cr_2_AlC^73^ and Ti_2_AlC^74^. The small energy dispersion along the K–H and L–M directions indicates the strong anisotropic behavior. In other words, the conductivity of MAX phase compounds is lower along the c-axis than to their basal planes. There is no apparent difference in band associated with Zr and Hf atoms in terms of energy level because electronegativity for Zr (1.33) and Hf (1.30) is almost the same. At different pressure, the increase in bandwidth was observed within the mentioned pressure limit. We found that with the increase in pressure from 0 to 50 GPa, the bands become looser, i.e., the bands above the Fermi level move upward while the bands below the Fermi level move downward. Moreover, the bands at the Fermi level increases with the increase in pressure.

**Figure 5:**
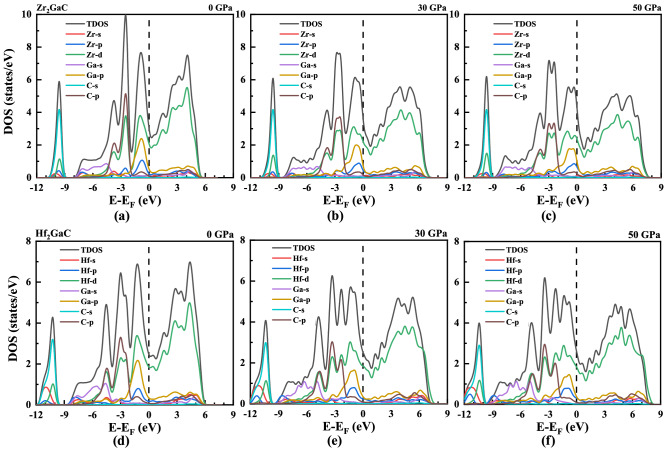
The total and partial density of states (PDOS) for Zr_2_GaC at pressure **a** 0 GPa, **b** 30 GPa, and **c** 50 GPa, and for Hf_2_GaC at pressure **d** 0 GPa, **e** 30 GPa, and **f** 50 GPa. Fermi level is set to 0 eV.

To explain the bonding behavior of M_2_GaC (M = Zr, Hf) MAX phases, we examine the density of states (DOS). The partial and total density of states (TDOS) for M_2_GaC MAX phase compounds at pressure 0 GPa, 30 GPa, and 50 GPa are depicted in Fig. 5. In our previous work^42^, the hybridization of M (4d, 4p, 5s), Ga (3d, 4p, 4s), and C (2p, 2s) orbitals for M_2_GaC is explained at 0 GPa. The TDOS at Fermi level at 0 GPa for Zr_2_GaC and Hf_2_GaC are 3.00 states/eV/unit and 2.47 states/eV/unit, respectively. In the Zr_2_GaC MAX phase, the lowest valance bands of TDOS is formed by C-s with Zr-d, Zr-p in the energy ranges from − 10.85 to − 9.11 eV. The states range from − 8.01 to − 5.1 eV, and − 5 to − 1.90 eV are formed by Ga-s states and strong hybridization of Zr-d and C-s states, respectively. The highest valance bands in the created by Zr-d and Ga-p hybridization, which is relatively weaker than of Zr-d and C-s states. These results are consistent with that of Cr_2_AlC MAX phase material, which has the maximum DOS at E_F_, i.e., 6.46 states/eV cell/unit^75^.

**Figure 6:**
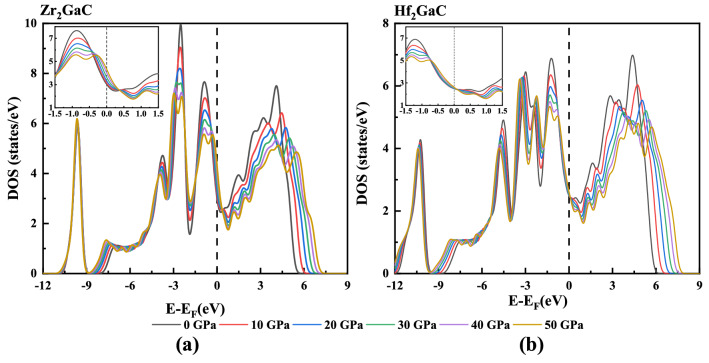
Total density of states (DOS) from GGA-PBE for M_2_GaC MAX phase (M = Zr, Hf) at different pressure. Figure inserted showing the effect of pressure in TDOS at Fermi level. Fermi level is set to 0 eV.

Moreover, the density of states at the Fermi level increased with increased pressure, and obtained results are tabulated in Table 1. It is observed that the increase in TDOS for the Zr_2_GaC MAX phase is more significant than that of Hf_2_GaC. The TDOS values for M_2_GaC illustrate that these MAX phase materials are metallic, and their metallicity is in the order of Zr_2_GaC > Hf_2_GaC in the given range of pressure. However, there is an increase in bandwidth, and correspondingly, the intensity decreased with the increase in pressure. The density of states on the right side of the Fermi level moves rightwards, whereas the density of states on the left side of the Fermi level moves leftwards under pressure (See Fig. 6), which is in good agreement with the analysis of band structure. It is worth noticing that the main contribution at the Fermi level is from the M-4d electrons in both MAX phases, which is not affected by the pressure. This implies that Zr-d and Hf-d electrons mainly contribute to the conduction properties of MAX phase materials under pressure.

**Figure 7:**
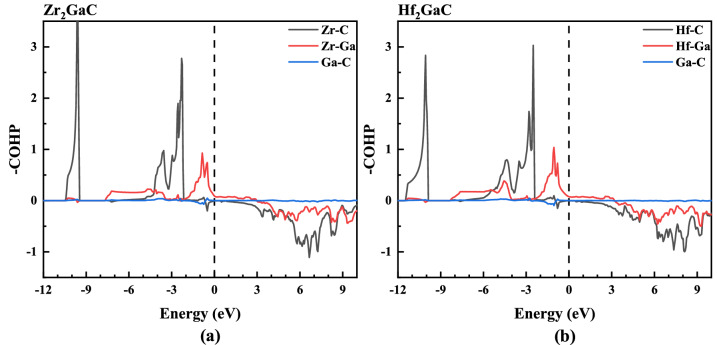
The calculated crystal orbital Hamilton population (COHP) showing the pair interactions of M–C, M–Ga, and Ga–C in **a** Zr_2_GaC and **b** Hf_2_GaC. Positive values in the y-axis (–COHP) indicating the bonding states. Fermi level is set to 0 eV.

To understand the chemical bonding of Zr_2_GaC and Hf_2_GaC MAX phase, we investigate the Crystal Orbital Hamilton Population (COHP) for M–C, M–Ga, and Ga–C bonds, respectively. The COHP method has been widely applied to investigate the bonding and antibonding analysis of many MAX phases^76–78^. Figure 7 shows the COHP at the ground state of Zr_2_GaC and Hf_2_GaC MAX phases. It is observed that the COHP curves for both MAX phases have well-defined bonding and antibonding regions with crossover points near the Fermi level, which is an indication of covalent bonding nature. There is no occupation of antibonding orbitals in the M–C bonds of Zr_2_GaC and Hf_2_GaC MAX phases. Moreover, the occupied region below the Fermi level is dominated by strong M–C interaction, while at the Fermi level, the M–Ga bonding character was dominant. There is almost zero bonding interaction for Ga–C atoms due to the absence of orbital overlapping. The COHP curves are similar to the most studied Al-containing MAX phases^77^. Furthermore, the calculated values for metallicity and Fermi energy in the pressure range 0–50 GPa are tabulated in Table 1. It is observed that the metallicity of both Zr_2_GaC and Hf_2_GaC phases increases with increasing pressure. According to Eq. (6), the conductivity mainly depends upon ${n \mathord{\left/ {\vphantom {n {v_{F} }}} \right. \kern-\nulldelimiterspace} {v_{F} }}$ ratio because *e, l,* and *m* are constants. In the given pressure range, the ${n \mathord{\left/ {\vphantom {n {v_{F} }}} \right. \kern-\nulldelimiterspace} {v_{F} }}$ ratio of M_2_GaC MAX phases is in order of $\left( {{n \mathord{\left/ {\vphantom {n {v_{F} }}} \right. \kern-\nulldelimiterspace} {v_{F} }}} \right)_{{Hf_{2} GaC}} > \left( {{n \mathord{\left/ {\vphantom {n {v_{F} }}} \right. \kern-\nulldelimiterspace} {v_{F} }}} \right)_{{Zr_{2} GaC}}$, hence we may conclude that the conductivity of Hf_2_GaC > Zr_2_GaC.

**Figure 8:**
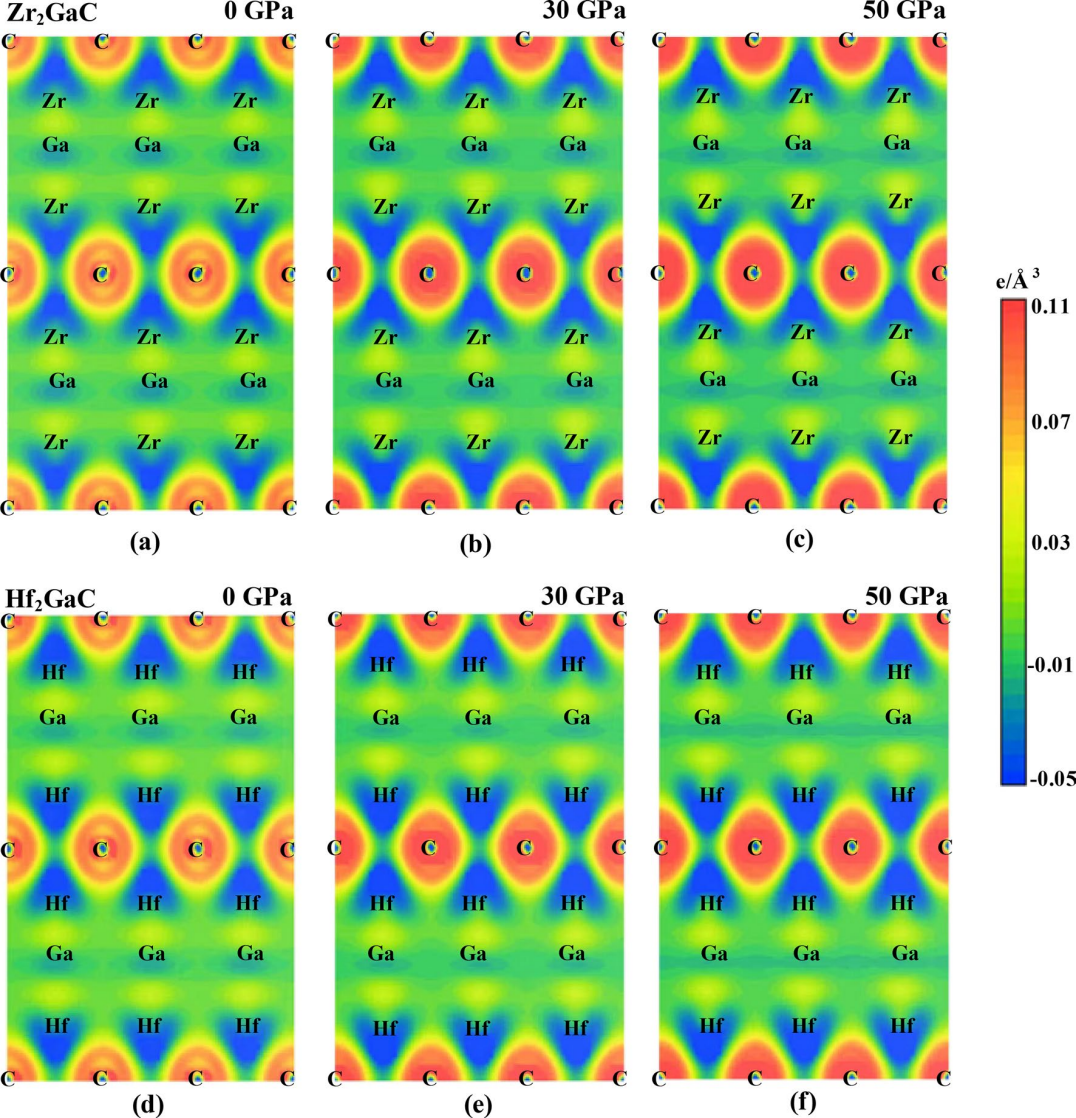
Electronic charge density mapping along the (100) plane for Zr_2_GaC at **a** 0 pressure, **b** 30 GPa, and **c** 50 GPa, and for Hf_2_GaC at **d** 0 pressure, **e** 30 GPa, and **f** 50 GPa.

To insight the chemical bonding of M_2_GaC MAX phases under pressure, the charge density distributions mapping along the (100) plane at 0 GPa, 30 GPa, and 50 GPa are plotted in Fig. 8. The bonding character of MAX phases is essential to understand the chemical bonding of their 2D derivatives (MXenes)^79,80^. The preferential accumulation of charges (positive regions at the scale bar) between two atoms indicates the covalent bonds, while balancing the positive or negative (depleted regions) charges at atomic position exhibits the ionic bonding^81^. At 0 GPa, the strong charge accumulation regions were observed at C and M = Zr, Hf atoms, indicating the formation of a strong covalent bond between C–Zr and C–Hf atoms. The charge accumulation at these atomic positions increases with an increase in pressure due to the decrease in atomic distance and an increase in internal parameters (z) (See Fig. 3). Furthermore, there is a sign of charge balancing around the Zr and Hf atoms, with the C indicating the small degree of ionic bonding. It is also seen that another covalent bond is formed between the Ga–M = Zr, Hf atoms, which is comparatively weaker than that of C–M = Zr, Hf atoms. Therefore, the chemical bonding in the M_2_GaC MAX phase is predicted to be a mixture of covalent and ionic nature and degree of bonding increases with the increase in pressure.

### Mechanical stability and dynamical properties

**Figure 9:**
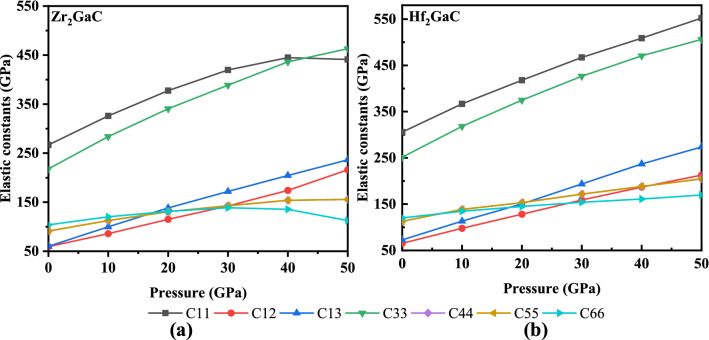
Elastic constants for M_2_GaC MAX phase (M = Zr, Hf) in function of pressure.

The mechanical properties of material help to predict the material’s response under the application of load. The mechanical properties of MAX phase materials also contribute to predicting the usefulness in service and are critical in the fabrication process. The elastic constants (C_ij_) for M_2_GaC MAX phase materials calculated in the pressure range from 0 to 50 GPa are shown in Fig. 9, and calculated mechanical properties are listed in Table 4. As we know that the M_2_GaC MAX phase has the hexagonal crystal structure, and there are six stiffness constants (C_11_, C_12_, C_13_, C_33_, C_44_ = C_55,_ and C_66_), but five of them are independent since $C_{66} = {{\left( {C_{11} - C_{12} } \right)} \mathord{\left/ {\vphantom {{\left( {C_{11} - C_{12} } \right)} 2}} \right. \kern-\nulldelimiterspace} 2}$^82^. Our results are consistent with other MAX phases.

**Table 4:** Variation of elastic constants and Vickers hardness in GPa for M_2_GaC (M = Zr and Hf) with pressure.

M_2_AX	Pressure	C_11_	C_12_	C_13_	C_33_	C_44_	B_v_	B_r_	G_v_	G_r_	B	G	H_v_	Ref
Zr_2_GaC	0	266	59	59	218	91	123	122	95	94			18.23	^42^
	10	325	85	99	283	112	167	167	112	111			16.77	
	20	377	115	138	340	130	208	208	125	124			15.47	
	30	419	142	171	388	142	244	244	134	132			14.28	
	40	444	174	204	436	153	276	276	138	136			12.63	
	50	440	216	236	462	155	302	301	128	124			9.27	
Hf_2_GaC	0	305	65	72	251	112	142	141	112	111			20.99	^42^
	10	366	97	113	318	138	188	188	130	129			19.36	
	20	417	128	150	374	153	229	229	142	140			17.56	
	30	467	159	193	426	171	272	272	153	150			16.14	
	40	508	186	236	470	188	312	312	162	157			14.84	
	50	552	212	273	506	204	347	347	172	164			14.20	
Zr_2_AlC		266	55	63	226	87					124	94	17.59	^69^
Zr_2_GaB		178	69	65	128	50					97	49	5.92	^85^
Hf_2_GaB		213	76	62	176	66					111	66	9.79	^85^
Ti_2_GaC		314	66	59	272	122					140	121		^28^
Ti_2_GaC		303	66	63	263	101					139	109		^86^

**Figure 10:**
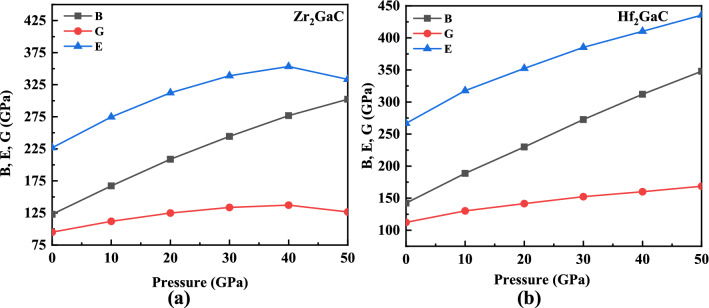
Variation of elastic moduli of M_2_GaC MAX phase (M = Zr, Hf) with pressure.

The elastic moduli versus pressure for the M_2_GaC MAX phase are plotted in Fig. 10. It is found that the elastic constants and moduli increase monotonically with an increase in pressure, and the values of C_11_, C_33_, Young’s modulus (E), and bulk modulus (B) increased significantly compared to other elastic constants. Contrary, the values of C_66_ and shear modulus (G) vary slowly. It can also be noticed that C_11_ and C_66_ for the Zr_2_GaC MAX phase increases uniformly up to 40 GPa and then decreases when pressure is exceeded to 50 GPa. A similar trend can be seen for Young’s and shear modulus of Zr_2_GaC. On the other hand, the Hf_2_GaC MAX phase shows a linear trend, consistent with the other MAX phase studies under pressure^55,83^. The mechanical stability of M_2_GaC MAX phases are predicted from the Born stability criteria^84^ i.e., C_11_ > 0, C_11_ − C_12_ > 0, C_44_ > 0, C_66_ > 0, (C_11_ + C_12_) C_33_ − 2$C_{13}^{2}$ > 0. Both MAX phases satisfy the mechanical stability criteria in the mentioned range of pressure. The elastic constant C_33_ for Zr_2_GaC and Hf_2_GaC increases by up to 462 GPa and 506 GPa, while the values for C_66_ for both materials increased only by 112 GPa and 169 GPa, respectively. The rapid increase in C_33_ and moderate C_66_ infers the increasing insensitivity of the compression strain along the c axis, not the shear strain.

It is known that the moduli (B, G, and E) measure the resistance of the material to fracture, plastic deformation, and stiffness and are essential to understand the solid-state properties, i.e., structural stability, ductility, stiffness, and brittleness. In this work, the elastic moduli (B, G, and E) increased with an increase in pressure from 0 to 50 GPa, i.e., for Zr_2_GaC increase in moduli are B (122–302 GPa), G (94–126 GPa), and E (226–333 GPa) and that of Hf_2_GaC are B (142–347 GPa), G (112–168 GPa), and E (266–435 GPa), respectively. The elastic moduli for the M_2_GaC MAX phase are in the order of $\left( {B,G,E} \right)_{{Hf_{2} GaC}} > \left( {B,G,E} \right)_{{Zr_{2} GaC}}$ in the given pressure range.

**Figure 11:**
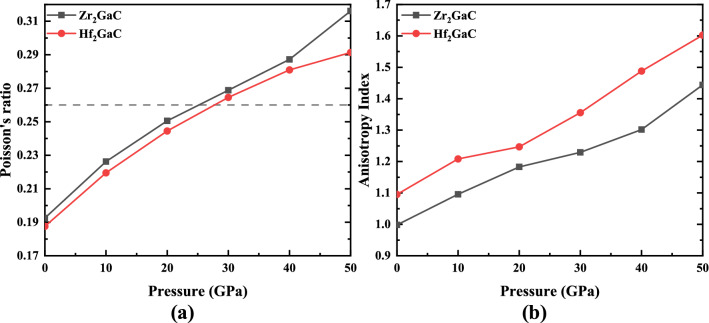
Variation of **a** Poisson’s ratio (σ), and **b** Anisotropic Index (A) for the M_2_GaC MAX phase (M = Zr, Hf) in function of pressure. The horizontal line in (**a**) is the proposed borderline between brittle and ductile transition.

The brittle/ductile behavior of the M_2_GaC MAX phase is predicted from the Poisson’s ratio (*σ*). It is the ratio between the transverse strain to longitudinal strain under tensile stress. It is an important tool to quantify the failure state in the solids. Frantsevich et al.^87^ proposed a borderline value *σ* ~ 0.26, which separates the ductile and brittle materials. For the brittle materials, this value is small, whereas the material is considered to be ductile if Poisson’s ratio is greater than 0.26. Our calculated values for *σ* for Zr_2_GaC and Hf_2_GaC are 0.192 and 0.187 at 0 GPa, respectively. These values increase linearly with an increase in pressure, as shown in Fig. 11a. There is a sharp increase in *σ* noticed for the Zr_2_GaC MAX phase when pressure increase from 40 to 50 GPa. In the pressure range mentioned, the M_2_GaC MAX phase behaves brittle manner at 0 GPa pressure, similar to many other MAX phases^28,88–90^. Both MAX phases exhibit ductile nature when pressure is increased from 30 GPa. Moreover, the microscopic hardness model proposed by Chen et al.^62^ was used to calculate the Vickers hardness of M_2_GaC MAX phases, and the obtained results are listed in Table 4. The theoretical Vickers hardness for (Zr_2_GaC)_Hv_ = 18.23 GPa < (Hf_2_GaC)_Hv_ = 20.99 GPa at 0 GPa pressure, respectively, and Vickers hardness decreases with an increase in pressure^69,85^. It is worth noticing that the grain size of the material has an essential effect on hardness, yield strength, tensile, and fatigue strength according to the Hall–Petch relation because grain boundaries hinder the movement of dislocations^91,92^. The effect grain size on the compressive strength of bulk Ti_2_AlC MAX phase followed the Hall–Petch relation under the dynamic and quasi-static loads^93^. Moreover, the oxidation resistance and mechanical properties of MAX phase thin films can be improved by increasing the grain boundaries^94,95^.

Figure 11b illustrates the anisotropic index (A) of the M_2_GaC MAX phase in the function of pressure. Typically, a material is called to be isotropic if anisotropic index A = 1 and the deviation from 1 indicate the anisotropic nature of the material. Figure 11b shows that the values of A do not satisfy the isotropic criteria, and an increase in pressure results in a higher anisotropic index for both Zr_2_GaC and Hf_2_GaC MAX phases suggesting the anisotropic nature of M_2_GaC MAX phases in the given pressure range. In other words, the properties for the M_2_GaC MAX is not identical in all directions. This fact is in good agreement with the anisotropic properties of M_2_GaC MAX phases, i.e., higher compressibility along the c-axis compared to other basal planes.

**Figure 12:**
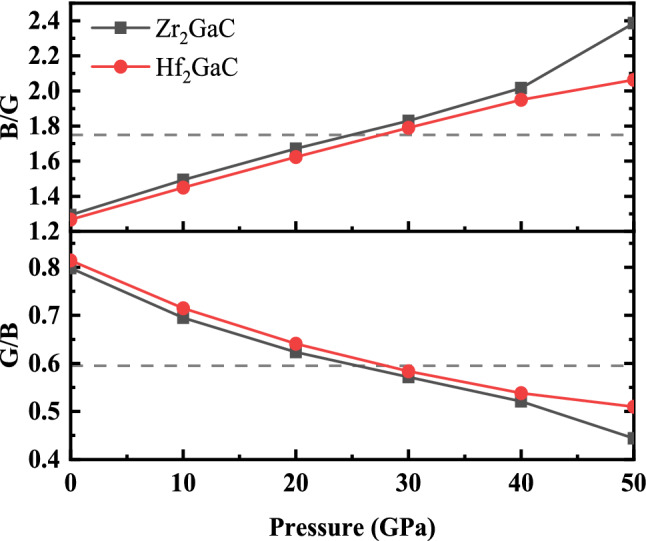
The effect of pressure on Pugh’s ratio for M_2_GaC MAX phase (M = Zr, Hf). The horizontal line in (**a**) is the proposed borderline between brittle and ductile transition.

According to Pugh’s criteria, a material will behave ductile if the B/G > 1.75 and G/B < 0.57, otherwise it should be brittle^62,96^. For 0 GPa these ratios for M_2_GaC MAX phases are in the order (Hf_2_GaC)_B/G_ = 1.26 < (Zr_2_GaC)_B/G_ = 1.29 and (Hf_2_GaC)_G/B_ = 0.78 > (Zr_2_GaC)_G/B_ = 0.77, respectively. This indicates that the M_2_GaC phases behave in a brittle manner at 0 GPa: however, with an increase in pressure, M_2_GaC phases likely to be ductile (See Fig. 12). These results are consistent with studies available in the literature^28^. It is also worth noticing that the ductility of Zr_2_GaC phase increase abruptly when pressure is exceeded from 40 GPa and pressure has significant impact in term of brittle/ductile transition for Zr_2_GaC compared to that of Hf_2_GaC. Moreover, the ratio G/B can also be used to determine the chemical bond. For ionic materials, the value is G/B $\approx$ 0.6, and that of covalent materials G/B $\approx$ 1.1. In this work, G/B values change from 0.77 to 0.41 for Zr_2_GaC and 0.78–0.48 for Hf_2_GaC, suggesting that ionic bonding is crucial for M_2_GaC compounds.

The Debye temperature is characteristic of solids that can evaluate many physical properties of the material, including thermal conductivity, thermal expansion, specific heat, and melting temperature. It can be calculated by numerous methods, among which the Anderson method is simple and widely used^63^. In this method, the average sound velocity (V_m_) is used to calculate the Debye temperature. The calculated Debye temperature and sound velocities for M_2_GaC MAX phases are listed in Table 5. It is noticed that the density and Debye temperature increases with increasing the pressure, and at a given pressure, the Debye temperature is always in the order of (Zr_2_GaC)$\theta$_D_ > (Hf_2_GaC)$\theta$_D_. The calculated melting point for the M_2_GaC MAX phase is tabulated in Table 5. The melting temperature for Zr_2_GaC and Hf_2_GaC MAX phases understudied are 1481 K and 1648 K at 0 GPa and increases with an increase in pressure. The higher melting temperature values indicate that M_2_GaC MAX phases are suitable for high-temperature applications.

**Table 5:** The computed density (ρ), transverse (V_t_), longitudinal (V_l_), mean (V_m_) sound velocities, and Debye’s temperature (${\uptheta }$_D_) for the M_2_GaC phase (M = Zr, Hf) at 0–50 GPa pressure.

M_2_AX	Pressure (GPa)	$\rho \,({\text{g/cm}}^{3} )$	*V*_*t*_ (.10^3^ m/s)	*V*_*l*_ (.10^3^ m/s)	*V*_*m*_ (.10^3^ m/s)	$\theta_{{\text{D}}}$ (K)	T_m_(K)	References
Zr_2_GaC	0	6.407	3.85	6.24	4.24	788	1481	^42^
	10	6.683	4.03	6.79	4.46	816	1757	
	20	7.235	4.15	7.20	4.60	843	1997	
	30	7.558	4.20	7.47	4.67	868	2195	
	40	7.852	4.18	7.65	4.66	892	2343	
	50	8.128	3.94	7.61	4.41	915	2371	
Hf_2_GaC	0	10.85	3.21	5.18	3.54	720	1648	^42^
	10	11.53	3.36	5.60	3.71	739	1931	
	20	12.09	3.42	5.88	3.79	757	2170	
	30	12.58	3.47	6.14	3.85	774	2395	
	40	13.02	3.50	6.35	3.90	791	2586	
	50	13.42	3.54	6.53	3.95	807	2769	
Nb_2_AlC	0	6.35	4.40	7.31	4.87	592	1800	^98^
Nb_2_CuC	0	7.68	2.56	5.52	2.89	356	1569	^98^
Hf_2_AlC	0	10.10	3.38	5.50	3.73	439		^39^
Hf_2_AlN	0	10.64	3.36	5.67	3.72	445		^39^
Zr_2_AlC	0	5.56	4.22	6.89	4.66	544		^37^

**Figure 13:**
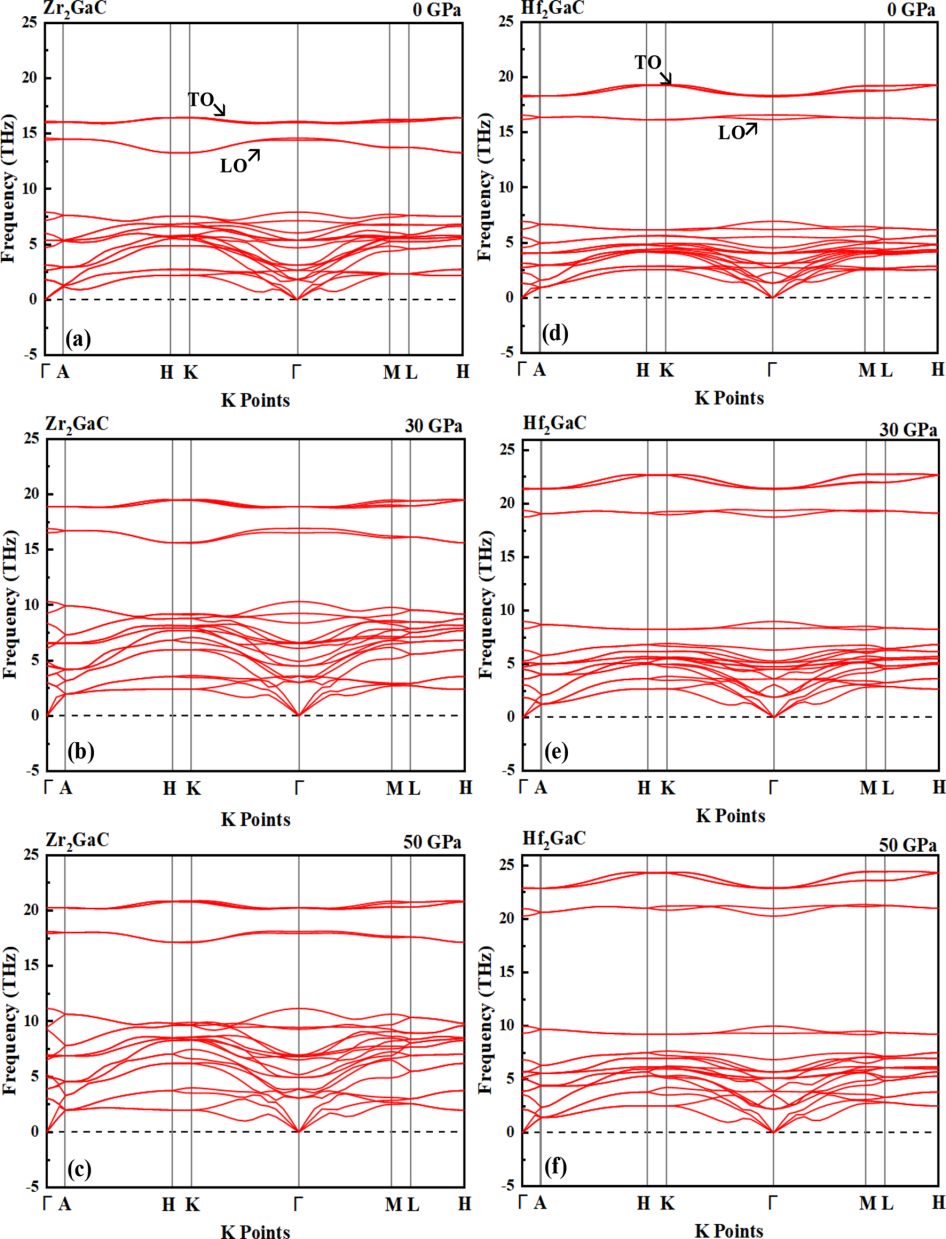
The phonon dispersion curve for Zr_2_GaC at pressure **a** 0 GPa, **b** 30 GPa, and **c** 50 GPa, and for Hf_2_GaC at pressure **d** 0 GPa, **e** 30 GPa, and **f** 50 GPa.

For the dynamical stability of M_2_GaC MAX phases, phonon calculation was performed along the high-symmetry directions in the Brillouin zone. The calculated phonon dispersion curves at 0 GPa, 30 GPa, and 50 GPa are shown in Fig. 13. There are eight atoms per unit cell in the 211 family of MAX phases. So, 24 phonon branches are produced; three are acoustic, and the rest are for optical modes. The optical branches are situated at the upper part of the dispersion curves, responsible for the optical behavior of MAX phase materials. These optical modes originate from the out-of-phase oscillations of atoms in lattice when one atom goes to the left and its neighbor to the right. In contrast, the acoustic branches are located at the lower part of phonon dispersion curves and arise from the coherent vibration of atoms in a lattice outside their balance position. The absence of negative frequencies in the phonon dispersion curves within the whole Brillouin zone robustly indicates the dynamical stability of M_2_GaC MAX phases under normal and high pressure against mechanical perturbation. The phonon dispersion curves become loose with the increase in pressure, which is consistent with band structures. At point Γ, the values of transverse optical (TO) and longitudinal optical (LO) frequencies and the separation between TO and LO increases with an increase in pressure. At 0 GPa, the values of LO (lower dispersion curve) and TO frequencies at Γ are 14.58, 16.56 THz for Zr_2_GaC and 16.00, 18.32 THz for Hf_2_GaC, respectively. Moreover, at the center zone point (Γ), the acoustic mode frequency is zero for all pressures, which is another indication of the stability of M_2_GaC MAX phases within the given pressure range.

**Figure 14:**
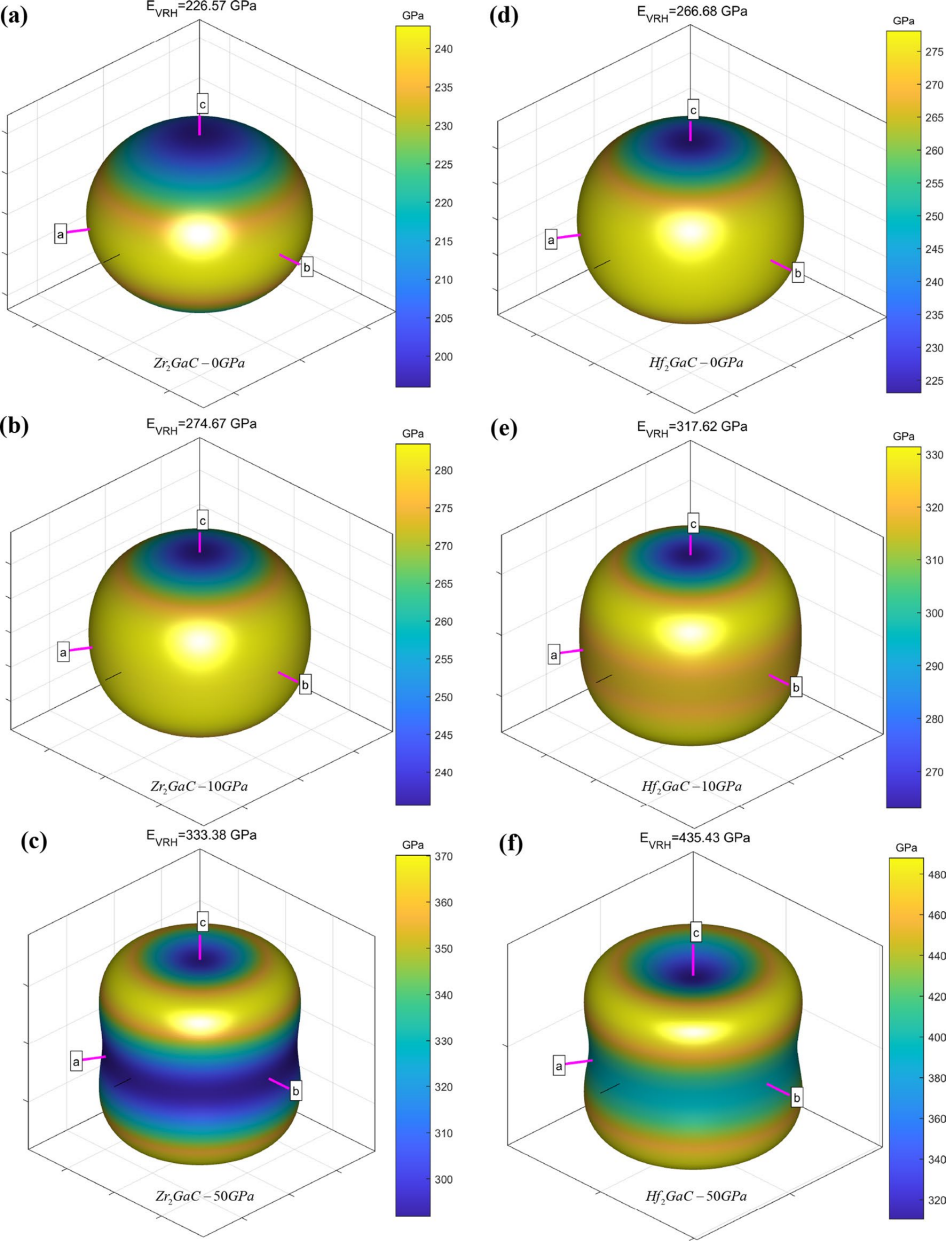
3D plot of Young’s modulus (E) surface of M_2_GaC MAX phase (M = Zr, Hf) at various pressure.

As mentioned earlier that the MAX phases compounds studied in this work have an elastic anisotropic nature, while the elastic anisotropy is not apparent. Figure 14 plots the 3D Young’s modulus surfaces obtained by an open-source software package (AnisoVis)^97^ of Zr_2_GaC (a, b, c) and Hf_2_GaC (d, e, f) at 0, 10, and 50 GPa, respectively. It can be observed that the shape begins to deviate from the sphere with an increase in pressure, and color varies in different regions, which indicates the elastic anisotropy of M_2_GaC MAX phases. The color variation (dark blue to yellow) exhibits that elastic modulus increases with an increase in pressure, and obtained values are mentioned at the top of each 3D plot, which agrees well with the previously calculated results (see Fig. 10). The Young’s modulus at 0 GPa pressure for Zr_2_GaC and Hf_2_GaC are 226.57 GPa and 266.68 GPa and rises to 333.38 GPa and 435.43 GPa when pressure is increased to 50 GPa, respectively. The pressure effect on the elastic anisotropy of Hf_2_GaC is more significant than that of the Zr_2_GaC MAX phase. According to the deviation degree of spherical shape, the anisotropy of Hf_2_GaC is bigger than Zr_2_GaC (See Fig. 11b).

### Optical properties

**Figure 15:**
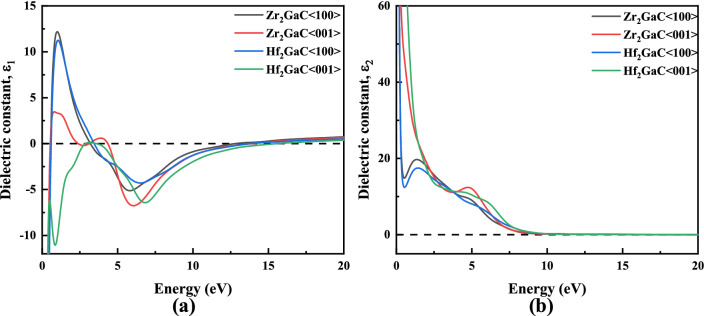
Energy dependence of dielectric function **a** real part **b** imaginary part of M_2_GaC MAX phase (M = Zr, Hf).

**Figure 16:**
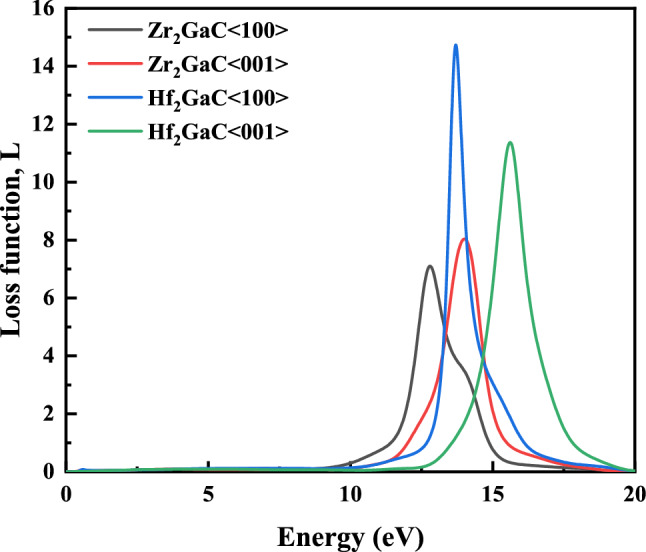
Loss function of M_2_GaC MAX phase (M = Zr, Hf) for <100> and <001> polarization.

**Figure 17:**
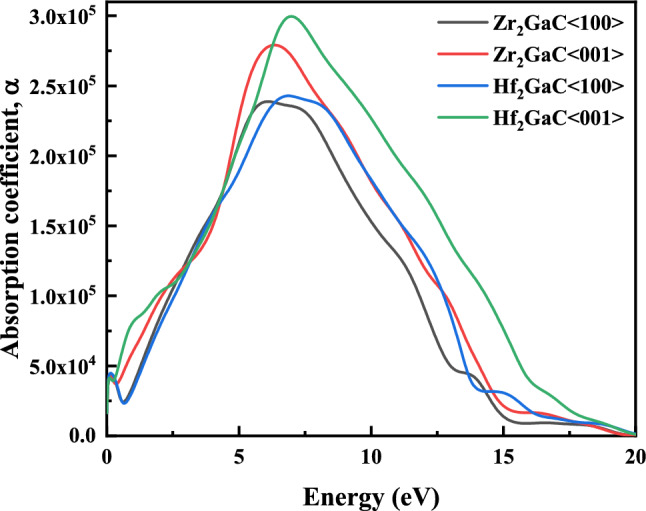
The optical absorption of M_2_GaC MAX phase (M = Zr, Hf) for <100> and <001> polarization.

**Figure 18:**
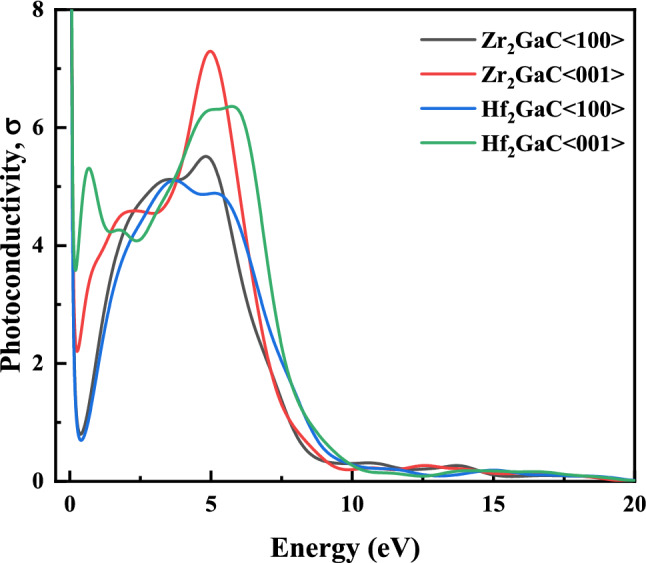
Photoconductivity of M_2_GaC MAX phase (M = Zr, Hf) for <100> and <001> polarization.

**Figure 19:**
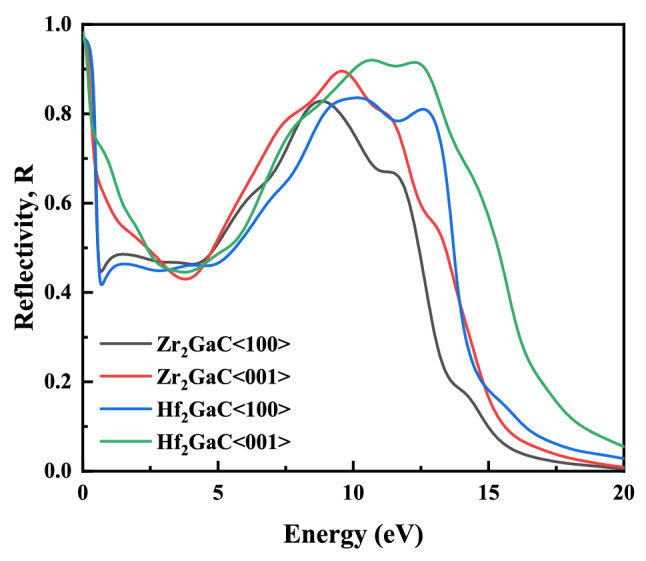
Reflectivity of M_2_GaC MAX phase (M = Zr, Hf) for <100> and <001> polarization.

The optical properties for the M_2_GaC MAX phase (M = Zr, Hf) were determined for the first time by frequency-dependent dielectric functions within the photon energies up to 20 eV. The optical properties for The MAX phases are optically anisotropic^99,100^. Thus, two polarization directions, <100> and <001>, were chosen to investigate the optical properties. The MAX phase compounds under this study are metallic in nature (see band structure) so, the term Drude (plasma frequency 3 eV and damping 0.05 eV) has been used with Gaussian smearing of 0.5 eV for all calculations. The frequency-dependent dielectric function $\varepsilon \left( \omega \right) = \varepsilon_{1} \left( \omega \right) + i\varepsilon_{2} \left( \omega \right)$(where $\varepsilon_{1} \left( \omega \right)$ is the real part and $\varepsilon_{2} \left( \omega \right)$ is the imaginary part of the dielectric function) has a close relation to the band structure. Once the imaginary part is known, the real part can be derived using the Kramers–Kronig equation. Later, all the optical properties can be obtained using the $\varepsilon_{1} \left( \omega \right)$ and $\varepsilon_{2} \left( \omega \right)$ ^101^, as shown in Figs. 15, 16, 17, 18 and 19.

#### Dielectric constant

The real part of the dielectric constant $\varepsilon_{1} \left( \omega \right)$ is essential for optoelectronic devices because it corresponds to the primitivity component that measures the stored energy. The imaginary part of the dielectric constant $\varepsilon_{2} \left( \omega \right)$ gives the information about the optical system's energy reduction in the function of frequency. Figure 15a,b shows the real $\varepsilon_{1} \left( \omega \right)$ and imaginary part $\varepsilon_{2} \left( \omega \right)$ of dielectric constants calculated for the < 100 > and < 001 > polarization directions for M_2_GaC. It is observed that the real part of dielectric constant ($\varepsilon_{1}$) approaches to zero from below, while the imaginary part ($\varepsilon_{2}$) gets to zero from above, which implies that the M_2_GaC MAX phases are metallic in nature^102^. In the real part of the dielectric constant, the spectra within the infrared region (I.R ≤ 1.7 eV) has the highest dielectric constant for <100> polarization compared to < 001 > polarization due to intra-band transition of electron. The sharp peaks were observed in $\varepsilon_{1} \left( \omega \right)$ for Zr_2_GaC and Hf_2_GaC phases along the <100> polarization at ∼1.01 eV and ∼1.04 eV, respectively. It is worth noticing that the spectra of $\varepsilon_{1} \left( \omega \right)$ for different polarization directions exhibit different features in the photon energy range. There is no significant difference observed for both MAX phases in the $\varepsilon_{2} \left( \omega \right)$ spectra for < 100 > and < 001 > polarization. Thus, we can deduce that the MAX phases studies here are optically anisotropic. Moreover, the value $\varepsilon_{1} \left( \omega \right)$ approached zero from below for polarization $< 100 >_{{M_{2} GaC}}$ at around 12.8–13.7 eV and for polarization $< 001 >_{{M_{2} GaC}}$, reaches zero at approximately 13.9–15.6 eV. While the value for $\varepsilon_{2} \left( \omega \right)$ reaches zero from above for polarization <100> and <001> at photon energy ranges from 12.5 to 16.7 eV.

#### Loss function

The loss function is peak corresponds to the bulk plasma frequency ($\omega_{p}$), which appears where $\varepsilon_{2}$ < 1 and $\varepsilon_{1}$ approaches to zero. It is the energy loss of the first electron traversing through a material, and bulk plasma frequency ($\omega_{p}$) is obtained from the loss function spectrum. The studied M_2_GaC MAX phases become transparent if the frequency of incident light is higher than that of plasma frequency. By analyzing, the energy-loss function peaks of Zr_2_GaC and Hf_2_GaC phases was occurred at around 12.79 eV and 13.7 eV, respectively, for <100> polarization: and corresponding 14.00 eV and 15.61 eV for <001> polarization as shown in Fig. 16. It is noticed that the plasma frequent ($\omega_{p}$) of M_2_GaC for the <001> polarization is larger than that for <100> polarization^103^. Moreover, energy loss spectra for M_2_GaC MAX phases show no peaks in the photon energy range of 0–10 eV due to large $\varepsilon_{2} \left( \omega \right)$ (see Fig. 15b).

#### Absorption coefficient

The absorption coefficient gives the knowledge about the efficiency of the solar energy conversion, which is important for solar cell material. It corresponds to the amount of light of a specific wavelength into a solid before getting absorbed. Figure 17 depicts the energy-based absorption (α) spectra of M_2_GaC MAX phases. It is observed that the absorption spectra for both MAX phases are weak in the infrared region (I.R), increases monotonously in the visible region and dominant ultraviolet (UV) regions. The maximum value of α was observed for Zr_2_GaC and Hf_2_GaC at around 6.09 eV and 6.86 eV, respectively, for <100> polarization; and corresponding 6.34 eV and 6.97 eV for <001> polarization. Moreover, the light absorption of M_2_GaC in the <001> polarization direction is larger than that for <100> polarization. In other words, both Zr_2_GaC and Hf_2_GaC MAX phases absorb more light in the direction of <001> polarization compared to its counterpart <100> , indicating their optically anisotropic nature^98^. The rise in α was observed in the direction of the UV region, exhibits the high absorbent feature of the material. Based on the calculated absorption spectra of M_2_GaC MAX phases, it can be deduced that these materials are competing candidates for optoelectronic devices in both visible and UV regions.

#### Photoconductivity

Photoconductivity (σ) of material can be described as the increase in the electric conductivity due to absorbing photos. For M_2_GaC MAX phases σ is shown in Fig. 18. It is noticed that for both MAX phases under this study, photoconductivity increases exponentially when the photo energy goes to 0 eV as expected for metals because there is no band gap present in the M_2_GaC MAX phases. A sharp dip in photoconductivity of Zr_2_GaC and Hf_2_GaC for <100> polarization was observed at 0.37 eV and 0.39 eV, and that for polarization <001> was observed at 0.26 eV and 0.20 eV, respectively^88^. Peak heights for different polarization are different at various photon energies, and Zr_2_GaC <001> gives the highest peak at 4.98 eV. It is concluded that M_2_GaC MAX phases are photoconductive at near I.R, visible, and UV regions.

#### Reflectivity

Finally, reflectivity spectra of M_2_GaC MAX phases for <100> and <001> polarization, as a function of incident light are demonstrated in Fig. 19. The reflectivity for M_2_GaC MAX phases shows the highest reflectivity in the I.R region and visible region ranges from 4.4 to 13.10 eV and then approaches zero for both phases in the incident photon energy ranges from 19 to 22 eV. However, it is worth noticing that the reflectivity is almost constant for <100> polarization of Zr_2_GaC and Hf_2_GaC MAX phases within the visible region, and values are above 45% and should appear as a metallic gray color. It is known that materials having constant reflectivity in the visible regions with an average value of about 44% are capable of reflecting the solar light, which results in a reduction in solar heating in the visible light region^104^. So, it may be concluded that Zr_2_GaC and Hf_2_GaC MAX phases can be used as the coating material for the purpose of solar heating reduction. However, the variable reflectivity within the visible region of different polarization indicated the optical anisotropy M_2_GaC MAX phase^105^.

**Figure 20:**
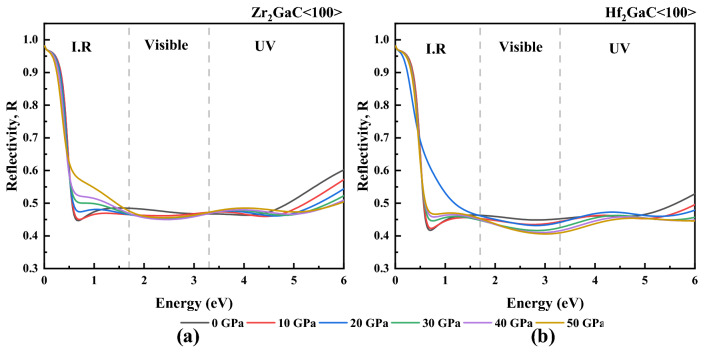
Reflectivity of M_2_GaC MAX phases (M = Zr, Hf) for <100> polarization at 0–50 GPa pressure.

The dependence of reflectance on the pressure of M_2_GaC MAX phases was studied as well, and results for polarization <100> at a pressure range from 0 to 50 GPa is shown in Fig. 20. For the M_2_GaC MAX phase, the reflectance exhibits less change in the moderate range of the I.R region ranging from 0 to 0.48 eV at all pressures and show variable reflectivity in the rest I.R region. It is noticed that the reflectivity increases with an increase in pressure in the I.R region. However, the reflectivity of M_2_GaC decreased at higher pressure, but almost the same in the visible region then increases in the UV region more quickly and exhibits a higher value at 0 GPa. The reflectance at pressure range 0–50 GPa remains above 40% in the visible zone. Thus, it is concluded that M_2_GaC MAX phase materials are ideal for coating materials under high-pressure conditions to avoid solar heating in the <100> polarization direction.

## Conclusion

The effect of pressure on structural stability, mechanical, electronic, phonon, and optical properties of M_2_GaC MAX phases (M = Zr and Hf) in the pressure range from 0 to 50 GPa were calculated by using first-principles calculations. The formation of enthalpy with respect to its most competing phases showed that M_2_GaC MAX phases are thermodynamically stable. The band structure and total density of states exhibited that M_2_GaC MAX phases are metallic in nature, with an increase in bandgap at the Fermi level with an increase in pressure. The DOS at E_F_ in the pressure range of 0–50 GPa are Zr_2_GaC = 2.96–4.27 states/eV/unit > Hf_2_GaC = 2.47–2.60 states/eV/unit, which implies that the metallicity of Zr_2_GaC increased more than that of Hf_2_GaC with increasing the internal pressure. According to COHP analysis, M–C bonds are stronger than that M–Ga in both Zr_2_GaC and Hf_2_GaC MAX phases. The volume ratio and lattice parameters Zr_2_GaC and Hf_2_GaC decrease with increasing pressure, and the compressibility of Zr_2_GaC is better than that of Hf_2_GaC. Besides, the normalized bond lengths show that crystals compressed more easily along the M–Ga (Zr, Hf) direction under pressure. The effect of pressure on the mechanical properties of M_2_GaC MAX phases is pronounced. Both the Zr_2_GaC and Hf_2_GaC MAX phases revealed the brittleness behavior at 0 GPa pressure and tended to ductile when pressure increased from 10 to 50 GPa. Moreover, there is a linear increase in elastic constants, elastic moduli, Poisson’s ratio, and a decrease in Vickers hardness was observed with the increase in pressure. The calculated Vickers hardness is found to be 18.23 GPa and 20.99 GPa for Zr_2_GaC and Hf_2_GaC, respectively. The phonon dispersion curves have confirmed the dynamical stability of compounds in the given pressure range. The optical properties of the MAX phase compound under this study reveals some interesting information. The absorption spectra of M_2_GaC increased to the maximum value in the visible region, and the UV region indicates its high absorbent capability and is suitable for optoelectronic devices in the visible and UV regions. Moreover, the reflectance curves show the constant values in the visible region with an average value above 44%. We conclude that these compounds can also be used as a coating material to avoid solar heating at even high pressure. To the author’s best knowledge, no study had been made to predict the mechanical, electronic, thermal, phonon, and optical properties of M_2_GaC MAX phases under pressure. Hence, these results can serve as a reference for future theoretical and experimental research.
